# Acute Static Stretching Results in Muscle-Specific Alterations amongst the Hamstring Muscles

**DOI:** 10.3390/sports8090119

**Published:** 2020-08-30

**Authors:** Manon Riccetti, Jules Opplert, Joao L. Q. Durigan, Carole Cometti, Nicolas Babault

**Affiliations:** 1 Centre for Performance and Expertise, INSERM UMR1093-CAPS, University of Burgundy Franch-Comte, Sport Science Faculty, F-21000 Dijon, France; Manon.riccetti@outlook.fr (M.R.); opplert.jules@gmail.com (J.O.); carole.cometti@u-bourgogne.fr (C.C.); 2 College of Physiotherapy, University of Brasılia, Brasilia 70910-900, Brazil; joaodurigan@gmail.com

**Keywords:** passive torque, biceps femoris, semitendinosus, mechanical properties, myotendinous junction

## Abstract

This study aimed to explore the acute effects of static stretching on the musculotendinous properties of two hamstring muscles. Twelve male volunteers underwent two testing sessions. One session was dedicated to the evaluation of the semitendinosus muscle before (PRE) and after (POST) static stretching (five sets of 30-s stretching), and the other session similarly explored the long head of biceps femoris muscle. In addition to the displacement of the myotendinous junction (MTJ), passive torque and maximal voluntary isometric torque (MVIT) were evaluated. MVIT (−8.3 ± 10.2%, *p* = 0.0036, d = 0.497) and passive torque (−28.4 ± 16.9%, *p* = 0.0003, d = 1.017) were significantly decreased POST stretching. PRE stretching, MTJ displacement was significantly greater for semitendinosus muscle than biceps femoris muscle (27.0 ± 5.2 vs. 18.6 ± 3.6, *p* = 0.0011, d = 1.975). After the stretching procedure, greater MTJ displacement relative changes were observed for biceps femoris muscle as compared to semitendinosus muscle (22.4 ± 31.6 vs. −8.4 ± 17.9, *p* = 0.0167, d = 1.252). Because of the smaller MTJ displacement PRE stretching and greater alteration POST stretching in biceps femoris muscles, the present study demonstrated muscle-specific acute responses of hamstring muscles during stretching. Although stretching acutely impairs torque production, the passive torque reduction and alteration of MTJ displacement might impact hamstring injury prevention.

## 1. Introduction

Static stretching is well known to alter the musculotendinous system and to produce significant increases in flexibility [1]. In contrast, when presented within a warm-up, static stretching could acutely induce several negative effects, including potential impairments of the contractile function as witnessed by decreases in voluntary strength [2,3]. These decreases mostly originate from alterations of (i) neuromuscular properties [4,5], and (ii) elastic properties of the muscle–tendon unit [6]. More particularly, musculotendinous stiffness decreases have been observed as a result of decreased passive torque and/or alterations within muscle fascicles, aponeuroses, and tendons [7,8].

Studies have shown that the acute effects of stretching on the musculotendinous system are dependent on various factors. Among them, recent studies have demonstrated that stretching-induced effects are dependent on the intrinsic stiffness of the musculotendinous system [9]. For instance, the authors proposed that the inter-individual variability in strength alteration following stretching was related to the initial flexibility [10]. It may partly be due to the different strain of muscles or tendons during stretching as a function of individuals’ relative compliance [11]. In addition to individual differences, inter-muscle differences have been observed [9,12]. For example, stretching-induced differences have been observed between triceps surae and quadriceps muscles [9]. The authors hypothesised that the architecture of the musculotendinous system was a crucial parameter. Indeed, the muscle–tendon length, and more particularly the ratio between fascicle length and aponeurotic-tendon tissue has been demonstrated to influence stiffness alterations [9,13].

The tendon-to-fascicle length ratio has been shown to reflect the compliance of the muscle–tendon unit and the degree of fascicle/sarcomere shortening during stretching [14]. Moreover, a greater ratio has been shown to correspond to compliant tendons [15]. However, in a study comparing acute stretching effects on muscles with various tendon-to-fascicle length ratio (triceps surae vs. quadriceps muscles), the authors acknowledged that different amplitudes of stretching could influence their results [9]. This experimental bias could be avoided by investigating muscles within the same muscle group. Such analysis has previously been conducted on triceps surae muscles [12]. Nevertheless, because the hamstring muscles have large intra- and inter-muscle variations [16,17] and are often implied in stretching routines, studies exploring this muscle group are of great interest. To date, previous studies using various techniques have investigated the effects of stretching on the hamstring muscles but revealed conflicting conclusions for muscle–tendon stiffness alterations [17,18,19,20]. Therefore, the present study aimed to explore the acute effects of stretching on the musculotendinous properties of two muscles from the hamstring muscle group. Specifically, we focused on the displacement of the myotendinous junction (MTJ) of the long head of biceps femoris and semitendinosus muscles. With respect to tendon lengths (longer tendons in semitendinosus muscles as compared to the long head of biceps femoris), we hypothesised that alterations would be greater in semitendinosus as compared to biceps femoris muscle.

## 2. Materials and Methods

### 2.1. Participants

Twelve male volunteers were recruited for this study. Their mean age ± standard deviation (SD), height, and body mass were 22.9 ± 0.9 years, 178.5 ± 6.0 cm, and 72.8 ± 7.3 kg, respectively. Volunteers were physical education students. All were regularly trained with at least three training sessions per week. They were track and field or team sports athletes. None reported lower limb injuries within the last three months and the last two years specifically for the hamstring muscles. During the duration of the study, they were instructed to refrain from intensive exercise. Before participation, they were fully informed about the purpose of the study and experimental procedure. All signed an informed consent form. The sample size was calculated a priori using GPower software (University of Trier, Trier, Germany) based on the effect size reported in a previous study [9] to satisfy a power > 90%. This study was conducted according to the declaration of Helsinki and approval was obtained from the committee on human research of the Sport Science faculty (CEP1812B).

### 2.2. Experimental Procedure

**Figure 1:**
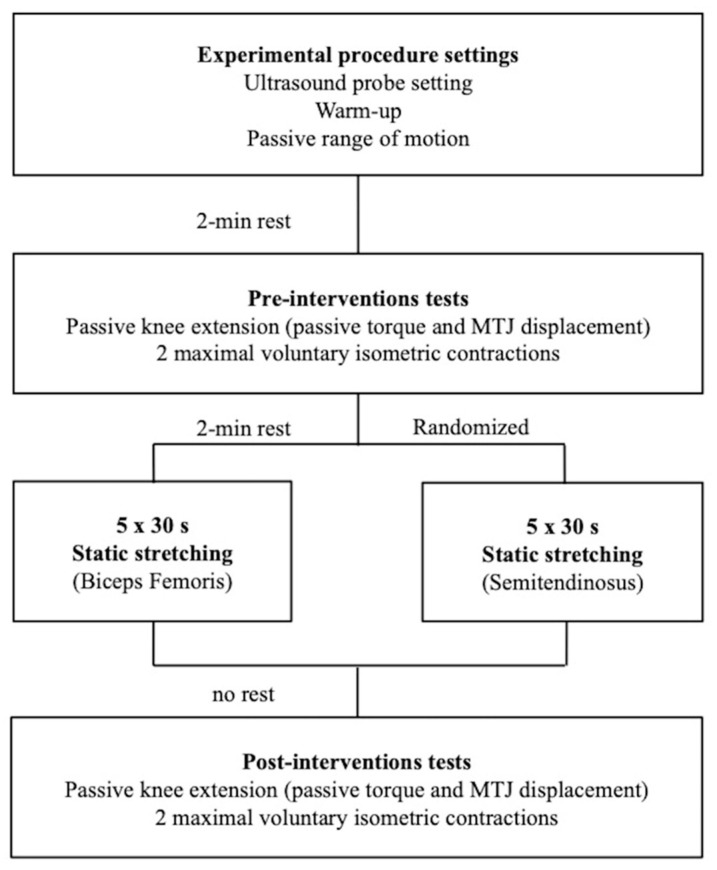
Timeline of the experimental protocol with the two randomised testing sessions. One session was dedicated to the investigation of the myotendinous junction (MTJ) of the biceps femoris muscle and the other for semitendinosus muscle.

Each participant came to the laboratory on three separate occasions. The first session was dedicated to the familiarisation (stretching procedure and tests) and to make initial anthropometric measurements. Then, one testing session was dedicated to the evaluation of the displacement of the myotendinous junction (MTJ) for semitendinosus and the other for biceps femoris muscles (Figure 1). The two experimental sessions were randomised, and a minimum of 24 h was imposed between sessions. Volunteers were tested at the same time of the day and were instructed to have similar daily activities and food intake before test sessions. All tests were performed on the right side.

**Figure 2:**
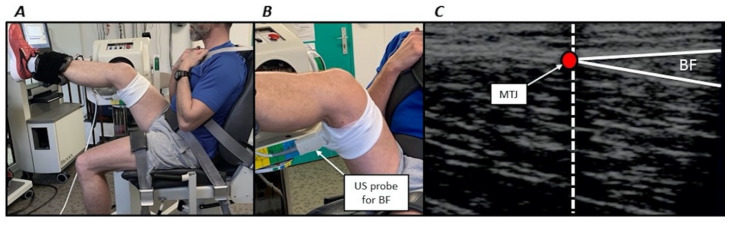
(**A**) volunteer positioning on the isokinetic dynamometer. (**B**) Zoomed image illustrating the ultrasonographic (US) probe positioning for the long head of biceps femoris muscle (BF). (**C**) Example of ultrasound image for the long head of biceps femoris muscle. The vertical dashed line coincides with the echo-intensive wire. The red circle (myotendinous junction, MTJ) corresponds to the junction of the superficial and deep aponeurosis.

During each session, participants were first seated on a Biodex isokinetic dynamometer (System 4, BIODEX Corporation, Shirley, NY, USA). They were secured with Velcro straps applied over the pelvis, trunk and contralateral thigh to the dynamometer to minimise inappropriate displacements. Then the right hip was flexed with a 45° angle between the thigh and torso. The left side was maintained with an approximately 100° hip angle. Then, the axis of the right knee joint (corresponding to the lateral condyle) was aligned with the input axis of the dynamometer according to the manufacturer’s guidelines. A dynamometer strap was firmly attached distally on the subject’s right thigh and a second one above the subject’s right ankle (Figure 2). An almost similar position has previously been used [3]. All stretch and torque measurements were performed using these participants’ positioning.

Then, participants conducted a brief warm-up composed of 10 knee extensions and flexions with increasing intensity. After this warm-up, the maximal passive range of motion (PROM) was determined using the isokinetic dynamometer. While doing a passive knee extension, the hamstring muscles were slowly stretched (5°·s^−1^) starting from a maximal knee flexion until the point of maximally tolerated discomfort but not pain. Then the leg immediately returned in a neutral position (90° knee flexion angle; 0° = complete knee extension). After two minutes of rest, a single passive stretch was performed using the dynamometer in a passive mode over the so-determined PROM in order to evaluate passive torque and MTJ displacements. A slow angular velocity (5°·s^−1^) was used in order to avoid the myotatic reflex and to obtain a proper resolution of ultrasonographic images. A constant-torque stretching procedure was used. Therefore, the maximal passive torque corresponding to the maximal PROM was registered and defined as a maximum tolerable torque threshold. This torque threshold was used during the static stretching procedures.

Immediately after, participants performed two maximal voluntary isometric contractions of the hamstring muscles in a neutral position (90° knee flexion). Contractions were 5 s duration long interspersed with 15 s rest. The single passive stretch and the two maximal voluntary isometric contractions served as pre-tests. Two minutes after these pre-tests (PRE), volunteers conducted the stretching procedure. Immediately after, post-tests (POST) were performed and replicated pre-tests in the same order (passive stretch until maximal PROM followed by two maximal voluntary isometric contractions).

Stretching included five sets of 30-s stretching using a constant-torque procedure [21]. During each stretch, the leg was passively rotated using the isokinetic dynamometer at slow angular velocity (5°·s^−1^), starting from a standardised knee flexion (neutral position = 90° of knee flexion) until the predetermined maximum tolerable passive torque threshold. This final position was maintained for 30 s. The leg then returned to the starting standardised position and stretch was repeated. The five 30-s stretches were performed with a 30-s rest period. Subjects were instructed to relax during the stretching, and not to offer any resistance to the dynamometer.

### 2.3. Measurements

Tests were conducted 2 min before (PRE), and immediately after (POST) the stretching procedure. Tests included (1) a single passive stretch to evaluate MTJ displacement and passive torque and (2) two maximal voluntary isometric contractions.

MTJ displacements were measured using a B-mode ultrasound video imaging (AU5, Esaote Biomedica, Florence, Italy) during a single passive stretch performed from the neutral position through the PROM. A 50-mm, 7.5 MHz linear array probe was oriented along the longitudinal axis of each muscle. MTJ was identified as the convergence of the deep and superficial aponeuroses (Figure 2). The probe was positioned at the distal MTJ of the long head of the biceps femoris muscle or semitendinosus muscle (depending on the randomisation). The probe was firmly attached over the skin in the pre-determined position using a custom-made apparatus that permitted a constant pressure from the probe to the dermal surface. An echo-intensive wire pasted over the skin was placed between the probe and the skin for reference [22]. This marker was used to check for a constant probe position and served as a reference to determine MTJ displacements (Figure 2). Sonography was recorded and synchronised with passive torque and knee joint angle using a custom-made trigger. MTJ position was calculated relative to the marker using an open-source measurement software (ImageJ, ver. 1.41, NIH, Bethesda, MD, USA) at the start of the stretch until PROM. Displacement was subsequently calculated as the MTJ position changes from the start of the stretch until PROM. Similar procedures are widely used in the literature [9]. Intraclass correlation coefficients with this procedure were 0.802 and 0.738 the long head of biceps femoris muscle and semitendinosus muscle, respectively (unpublished observations).

Torque and knee joint angle were recorded using a Biopac MP150 system (Biopac System, Santa Barbara, CA, USA). Passive torque was measured during passive stretches from the start of the stretch until PROM. Passive torque changes were subsequently calculated as changes from the start of the stretch until the PROM [6]. During maximal voluntary isometric contractions, only the best knee flexion was retained for analyses. The peak maximal voluntary isometric torque (MVIT) was measured.

### 2.4. Statistical Analyses

Statistical analyses were conducted using Statistica (version 8.0, Statsoft, Tulsa, OK, USA). The normality of all data was tested and confirmed by the Shapiro–Wilk test. A two-way mixed model analysis of variances (ANOVA) with repeated measures was performed. For MVIT and passive torque, factors were “session” (first and second testing session) and “time” (PRE vs. POST). For MTJ displacement, factors were “muscle” that corresponded to the comparison between biceps femoris and semitendinosus muscles and “time” for the comparison between PRE vs. POST values. Tukey post-hoc tests were conducted if significant main effects or interactions were present. Partial eta squared (partial η^2^) was calculated from the ANOVA. Values of 0.01, 0.06, and above 0.14 were considered as small, medium and large differences, respectively [23]. A Student *t*-test was also performed on percentage changes between PRE and POST to observe differences between sessions and muscles. Subsequently, qualitative descriptors of standardised effects were used for pair-wise comparisons with Cohen’s d < 0.5, 0.5–1.2 and >1.2 representing small, medium and large magnitudes of change, respectively [23]. *p* < 0.05 was taken as the level of statistical significance for all comparisons. Absolute values are expressed as mean ± SD and relative changes as percentages of pre-intervention tests ± SD. Confidence intervals of 95% (95% CI) are also presented. Finally, coefficients of variation (%) were calculated on PRE values.

## 3. Results

Coefficients of variation were first calculated on PRE values for MVIT, passive torque and MTJ displacement. Values obtained during the biceps femoris session and semitendinosus sessions were respectively 17.6% and 18.6% for MVIT, 34.1% and 27.9% for passive torque changes and 19.3% and 19.3% for MTJ displacement.

Results from the ANOVA are presented in Table 1 and values for MVIT, passive torque changes and MTJ displacement are presented in Table 2. The MVIT only revealed a main time effect. The post-hoc test indicated a significant MVIT decrease immediately after stretching (*p* = 0.0036, d = 0.497, small, −8.3 ± 10.2%). Similarly, passive torque changes only revealed a significant main time effect. The post-hoc test indicated a significant passive torque decrease immediately after stretching (*p* = 0.0003, d = 1.017, medium, −28.4 ± 16.9%).

Considering MTJ displacement, the mixed model ANOVA revealed a significant main muscle effect and muscle × time interaction. The post-hoc analysis performed on the interaction revealed that MTJ displacement was significantly greater for semitendinosus muscle than biceps femoris muscle at PRE (*p* = 0.0011, d = 1.975, large). No difference was observed between muscles after the stretching procedure (*p* = 0.5341, d = 0.492, small). Percentage changes between PRE and POST measurements were significantly different when comparing both muscles. Greater changes were observed for biceps femoris muscle than semitendinosus muscle (*p* = 0.0167, d = 1.252, large).

## 4. Discussion

The purpose of this study was to explore the acute effects of static stretching on musculotendinous properties of two muscles from the hamstrings. More particularly, we tested the hypothesis that stretching-induced effects are dependent on tendon length. As often registered after long-duration (>60 s) static stretching, our study first revealed a reduction in maximal voluntary torque [24] and passive torque [6]. Considering myotendinous displacement, our hypothesis was partly confirmed since greater MTJ displacement was registered in semitendinosus muscle as compared to the biceps femoris muscle before the stretching procedure but not after (after repeating five sets of 30-s).

Our results first demonstrated similar passive torque variations during the two experimental sessions reflecting good reproducibility of the stretching procedure. After stretching, passive torque was significantly reduced. Such alterations are commonly obtained in the literature [6,7,22,25,26,27,28]. It could originate from different structures within muscles or tendons. The contribution of these two structures has previously been investigated on different muscle groups with inconclusive findings [28]. Although the present study focused on the MTJ displacements, one cannot exclude the contribution of muscle stiffness on the passive torque alterations registered here.

During PRE tests, we observed a significantly greater displacement of the MTJ in semitendinosus muscle as compared to the long head of the biceps femoris muscle. This finding confirmed our primary hypothesis and also confirmed previous conclusions pointing out inter-muscle differences [9,12]. For example, larger MTJ displacements were observed for the gastrocnemius medialis as compared to rectus femoris muscles [9]. One explanation for the longer MTJ displacement during the first stretch in semitendinosus muscle as compared to biceps femoris could originate from different muscle and tendon lengths between muscles. It has previously been demonstrated that the semitendinosus muscle has longer fascicles and longer tendons than the long head of biceps femoris muscle [16]. Moreover, it is well known that longer tendons can account for a greater part of the motion of the entire muscle–tendon unit [29]. A single stretch would more likely impact MTJ displacement in semitendinosus muscle than in the long head of biceps femoris. Such behaviour has previously been observed in various muscles or muscle groups during stretching [9] or during other tasks such as plyometric jumps [30]. The longer the tendinous tissue, the greater the lengthening and, therefore, the greater the MTJ displacement.

In addition, with respect to a buffer mechanism [30], this longer semitendinosus MTJ displacement could arise from a greater intrinsic muscle stiffness (i.e., fascicle) as a result of architecture, fibre-type composition or muscle activation. Firstly, muscle architecture is widely different within these two muscles. Various studies using different techniques (cadavers and/or ultrasonography) registered very short fascicles in the long head of biceps femoris (half shorter) as compared to semitendinosus muscle [16,31]. For example, fascicles obtained from cadavers were 7.6 cm for the long head of biceps femoris and 18.6 cm for semitendinosus muscle [16]. In a previous study, longer fascicles were associated with greater fascicle elongation and shorter MTJ displacement during a single stretch [9]. Accordingly, one would speculate greater fascicles elongation in semitendinosus muscle and, therefore, greater compliance within this muscle. However, previous studies have also demonstrated non-uniform stiffness values within muscles before stretching that tend to become more uniform after stretching [32]. Further studies are clearly needed to explore the interaction between fascicles and tendons during stretching.

Moreover, previous authors [20] pointed out the importance of aponeurosis, which is longer in the long head of biceps femoris than semitendinosus muscle. Considering that aponeuroses could be stiffer than tendons [33], this tissue could significantly impact MTJ displacement. For example, some authors registered greater tendon-aponeurosis displacements following a passive knee extension (from 90° knee flexion to complete knee extension) in biceps femoris muscle than in semitendinosus muscle [20], a result in contrast with the shorter muscle–tendon unit elongation previously recorded for the same muscle as compared to semitendinosus muscle [18,19]. It should also be noted that the opposite results obtained between the present study and previous findings [20] could be related to the different hip angles used (hip flexed vs. straight, respectively). A greater hip angle would favour hamstring stretching as shown in biceps femoris fascicles or shear modulus [17,34]. Future studies should, therefore, concomitantly focus on the different tissues to better explore acute and chronic stretching effects.

Secondly, fibre-type content seemed unlikely to explain our results. Indeed, the two muscles under investigation appeared to have almost similar type II fibres proportion. A pioneering study obtained 55.2% and 54.6% type II fibres in the proximal portion of the long head of biceps femoris muscle and semitendinosus muscle, respectively [35]. Thirdly, unexpected muscle activation during stretching could acutely and slightly increase fascicle stiffness. Although not registered here, special attention was given to control and to remember volunteers to stay relaxed during the whole stretching manoeuvres.

Interestingly, the stretching procedure has different effects on muscle–tendon depending on the muscle considered. Changes after the stretching procedure (as compared to PRE) are greater in the biceps femoris muscle as compared to the semitendinosus muscle. Such a result was in apparent contradiction with our primary hypothesis but confirmed previous data. For example, a greater MTJ displacement elongation has been registered after a similar short stretching procedure (five sets of 30 s) in vastus lateralis muscle as compared with gastrocnemius medialis muscle (vastus lateralis muscle showing smaller displacement before the stretching procedure) [9]. This result confirmed previous findings that acute stretching effects are muscle-specific (muscles or even muscles’ portions under investigations) after repetitive stretching [20,32]. For example, the authors previously observed a non-uniform stiffness within gastrocnemii muscles that became more-homogeneous after static stretching [32]. These authors observed greater stiffness changes in the stiffest muscle portions. Taken as a whole, it confirmed the hypothesis that repetitive stretching would primarily affect stiff tissues [11,12].

The greater impact of stretching on biceps femoris MTJ displacement has large impact during training and, particularly, for the hamstring injury prevention. It has been shown that the long head of biceps femoris is predisposed to injury as a result of its architecture [36]. This muscle has been shown to undergo the greatest lengthening during sprints. However, with shorter fascicles, and therefore reduced extensibility, repetitive over-lengthening is frequent. According to our results, it can be speculated that the longer MTJ displacement after stretching would compensate for the reduced fascicles extensibility. However, other factors (such as aponeuroses [37]) are also involved in injuries and should be taken into account. Stretching the hamstring muscles before explosive-type activities such as sprinting appear essential in order to decrease stiffness and, therefore, likely reduce injury risks. Moreover, flexed hips are essential to stretch muscle–tendon structures more efficiently [17,34].

However, one should keep in mind that acute stretching effects are often accompanied by an impairment of the contractile function. The present study registered a small but significant maximal voluntary torque reduction as previously obtained [38,39]. Exploring the mechanisms behind this torque reduction is beyond the scope of the present study, but both muscular and neural mechanisms are generally implied [4,40]. Because force losses after stretching are dose-dependent, coaches should pay attention to adequate program static stretching in order to optimise the balance between flexibility gains for injury prevention and the detrimental force losses.

There are several limitations to this study. Firstly, the reliability of our results could be questioned. We previously quantified moderate-to-good reliability of our measurements (unpublished observations). Nevertheless, values appeared notably lower than those obtained in more often studied muscles such as gastrocnemius muscles [13]. Secondly, large variability was obtained between volunteers. Testing a larger sample would increase the statistical power and consequently the significance of our results. Thirdly, the present study focused on one main parameter. Considering others such as fascicle length or pennation angle would have been of interest to determine differences between muscular and tendinous tissue alterations and to limit potential speculations. Considering other parameters and other muscles in future studies would allow us to generalise our findings.

## 5. Conclusions

In conclusion, the present study demonstrated different MTJ displacements amongst hamstring muscles (semitendinosus vs. biceps femoris muscles) during a single stretch. The greatest MTJ displacements were observed in semitendinosus muscle. Moreover, acute stretching effects were also muscle specific. Greater acute stretching effects were observed on stiffer tissues, i.e., biceps femoris muscle. Studies exploring musculotendinous properties should pay attention to the muscle history (whether it is a single isolated stretch or after repetitive stretching). Although stretching acutely impairs torque production, our results justify the use of static stretching in the hamstring muscles for injury prevention.

## Figures and Tables

**Table 1:** Results from the mixed-model ANOVA before (PRE) and after (POST) stretching interventions.

Parameter	Effect	F	*p*	Partial η^2^	Power
MVIT	Session	0.955	0.3493	0.079	0.145
	Time	13.553	0.0036 *	0.552	0.917
	Session × Time	2.033	0.1816	0.156	0.256
Passive torque	Session	2.832	0.120	0.204	0.336
	Time	25.613	0.0003 *	0.699	0.996
	Session × Time	4.796	0.051	0.303	0.515
MTJ displacement	Muscle	10.803	0.0072 *	0.495	0.848
	Time	0.205	0.659	0.018	0.069
	Muscle × Time	8.260	0.0151 *	0.429	0.744

MTJ, myotendinous junction; MVIT, maximal voluntary isometric torque. *: *p* < 0.05.

**Table 2:** Maximal voluntary torque, passive torque and myotendinous junction displacement before (PRE) and after (POST) stretching interventions.

Parameter	Session	PRE	POST	% Change
MVIT (N.m) *	Biceps femoris	106.3 ± 18.7	100.0 ± 19.9	−5.9 ± 10.3
	(95% CI)	(94.4; 118.2)	(87.3; 112.6)	(−12.4; 0.7)
	Semitendinosus	114.7 ± 21.7	101.7 ± 19.6	−10.7 ± 10.5
	(95% CI)	(100.9; 128.5)	(89.2; 114.1)	(−17.4; −4.1)
Passive torque (N.m) *	Biceps femoris	46.3 ± 15.8	29.6 ± 12.8	−34.8 ± 18.2
	(95% CI)	(36.3; 56.4)	(21.5; 37.8)	(−46.4; −23.2)
	Semitendinosus	45.9 ± 12.8	35.8 ± 12.4	−22.1 ± 14.4
	(95% CI)	(37.7; 54.0)	(27.9; 43.7)	(−31.2; −13.0)
MTJ displacement (mm)	Biceps femoris	18.6 ± 3.6	22.1 ± 4.8	22.4 ± 31.6
	(95% CI)	(16.2; 20.9)	(19.1; 25.2)	(2.3; 42.5)
	Semitendinosus	27.0 ± 5.2 †	24.3 ± 4.3	−8.4 ± 17.9 †
	(95% CI)	(23.7; 30.3)	(21.5; 27.0)	(−19.7; 2.9)

MVIT, maximal voluntary isometric torque; MTJ, myotendinous junction. Values are means ± SD and 95% confidence intervals (95% CI). *: significant time effect (*p* < 0.05), †: significant differences between biceps femoris and semitendinosus muscle for the same time point (*p* < 0.05).
